# Supplementary figures and images for: DoAP2/ERF89 activated the terpene synthase gene DoPAES in Dendrobium officinale and participated in the synthesis of β-patchoulene
Source: PeerJ. 2024 Jan 18;12:e16760. doi: 10.7717/peerj.16760 (PMC10800100; doi:10.7717/peerj.16760)

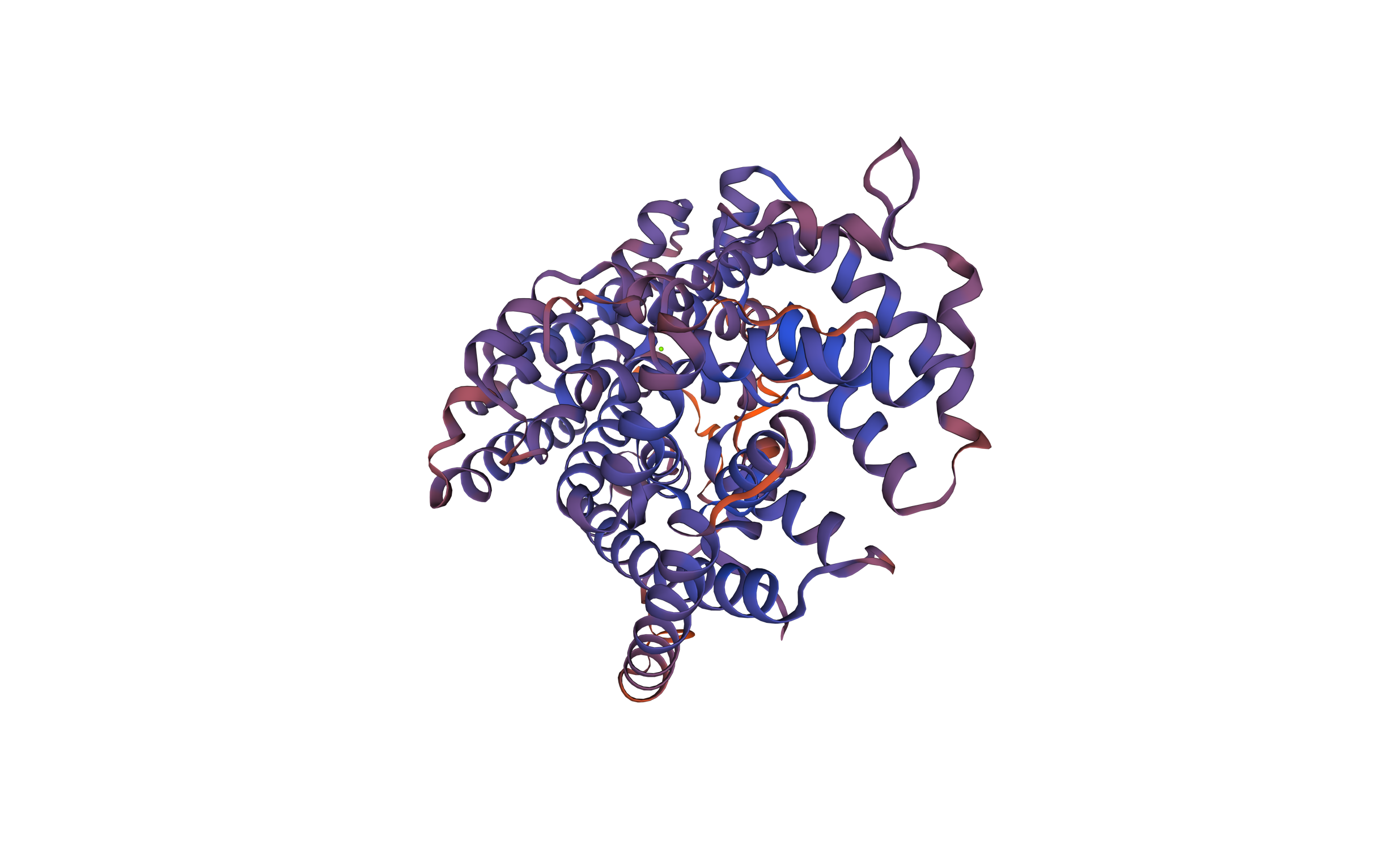

Supplement: Supplemental Information 1 — The protein template is sesquiterpene synthetase, and the conformation of DoPAES is similar to that of terpene synthetase of other species. [file peerj-12-16760-s001.png]

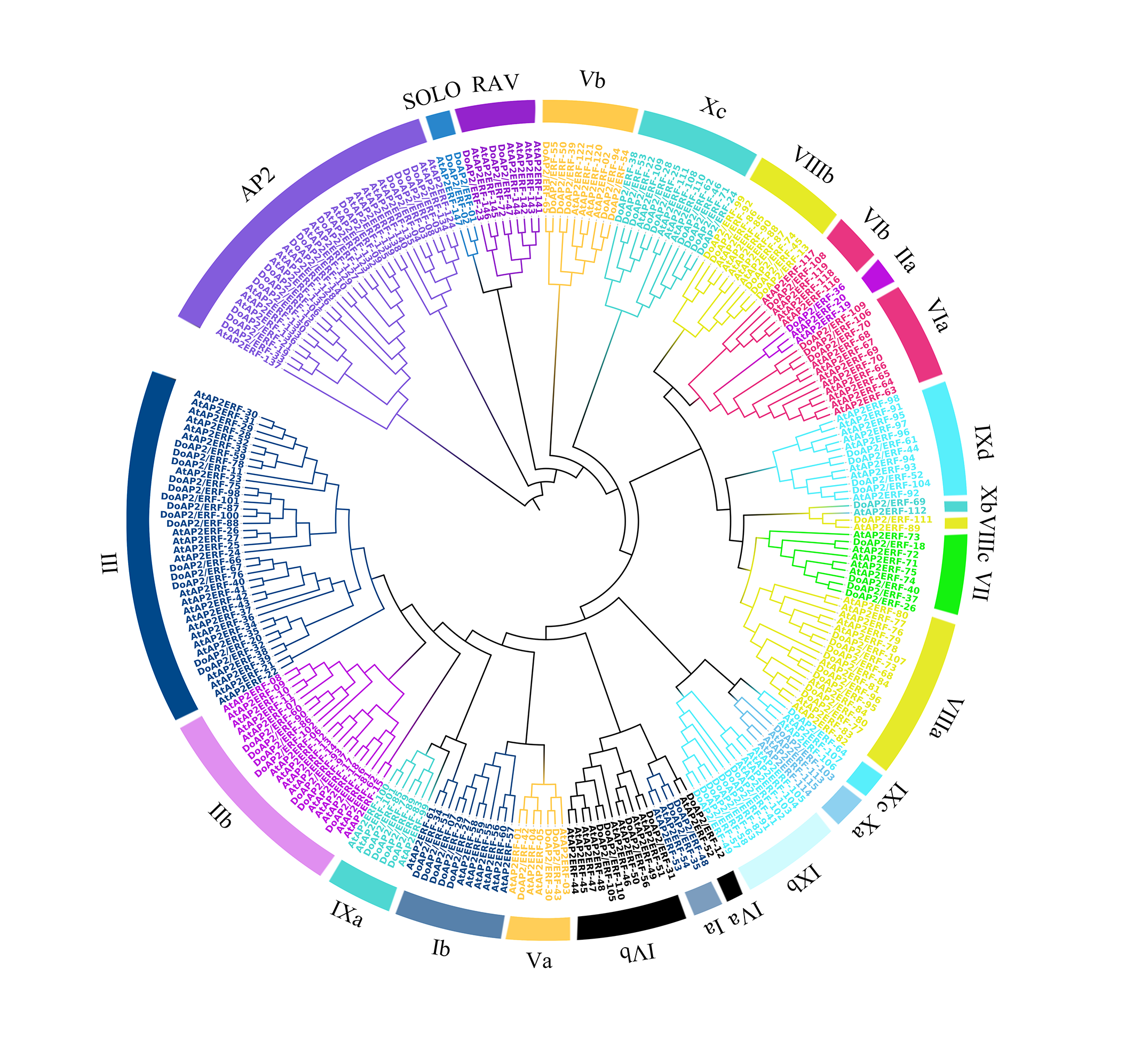

Supplement: Supplemental Information 2 — In total, 111 AP2/ERF proteins from D. officinale, 147 AP2/ERF proteins from A. thaliana were selected to construct the tree. Ⅰ–Ⅹ indicated the divided subfamilies according to the categories of AP2/ERF proteins in A. thaliana. [file peerj-12-16760-s002.png]

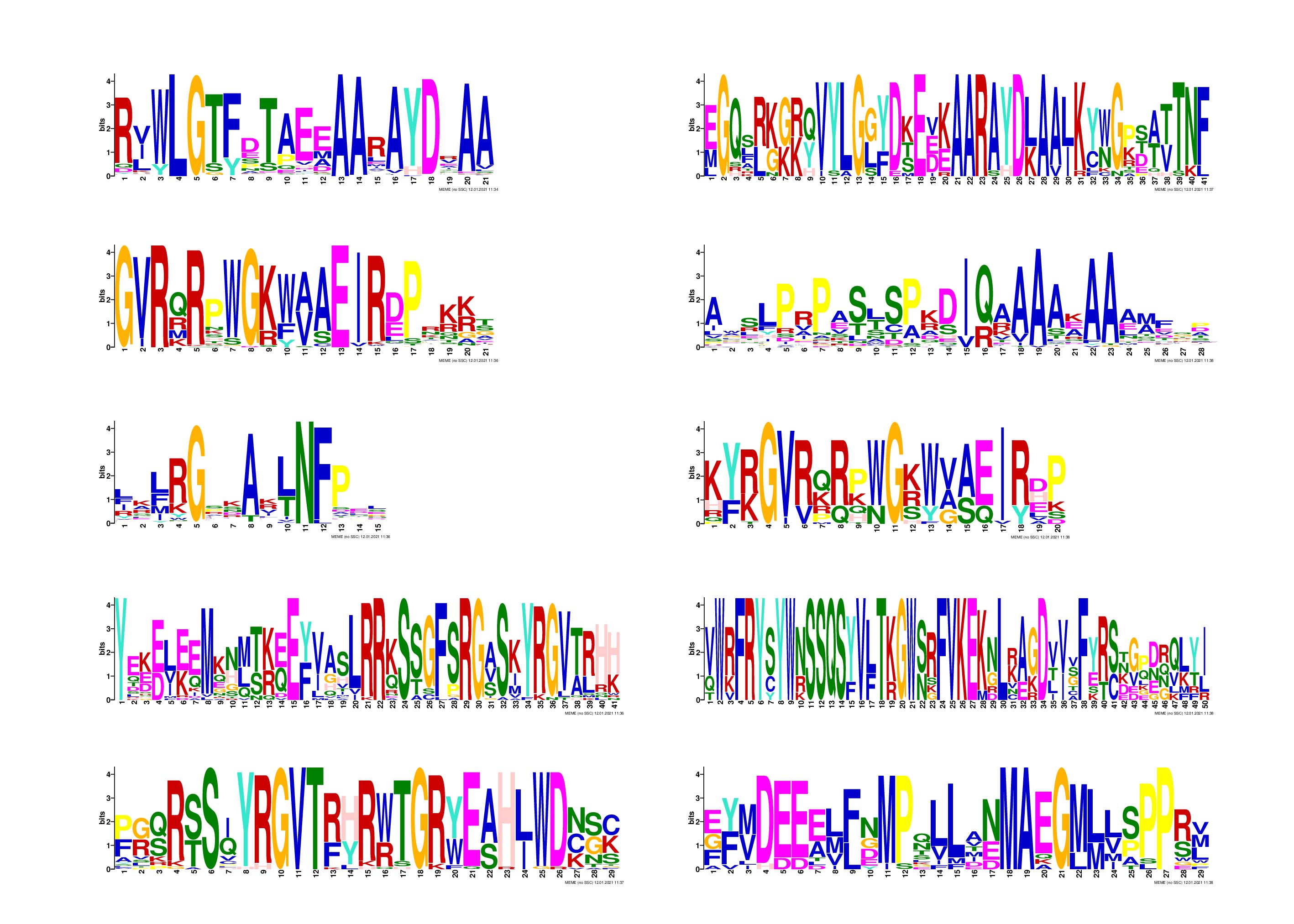

Supplement: Supplemental Information 3 — Motif 1–10 represents identity based on multiple alignment analysis of 111 DoAP2/ERF proteins. [file peerj-12-16760-s003.jpg]

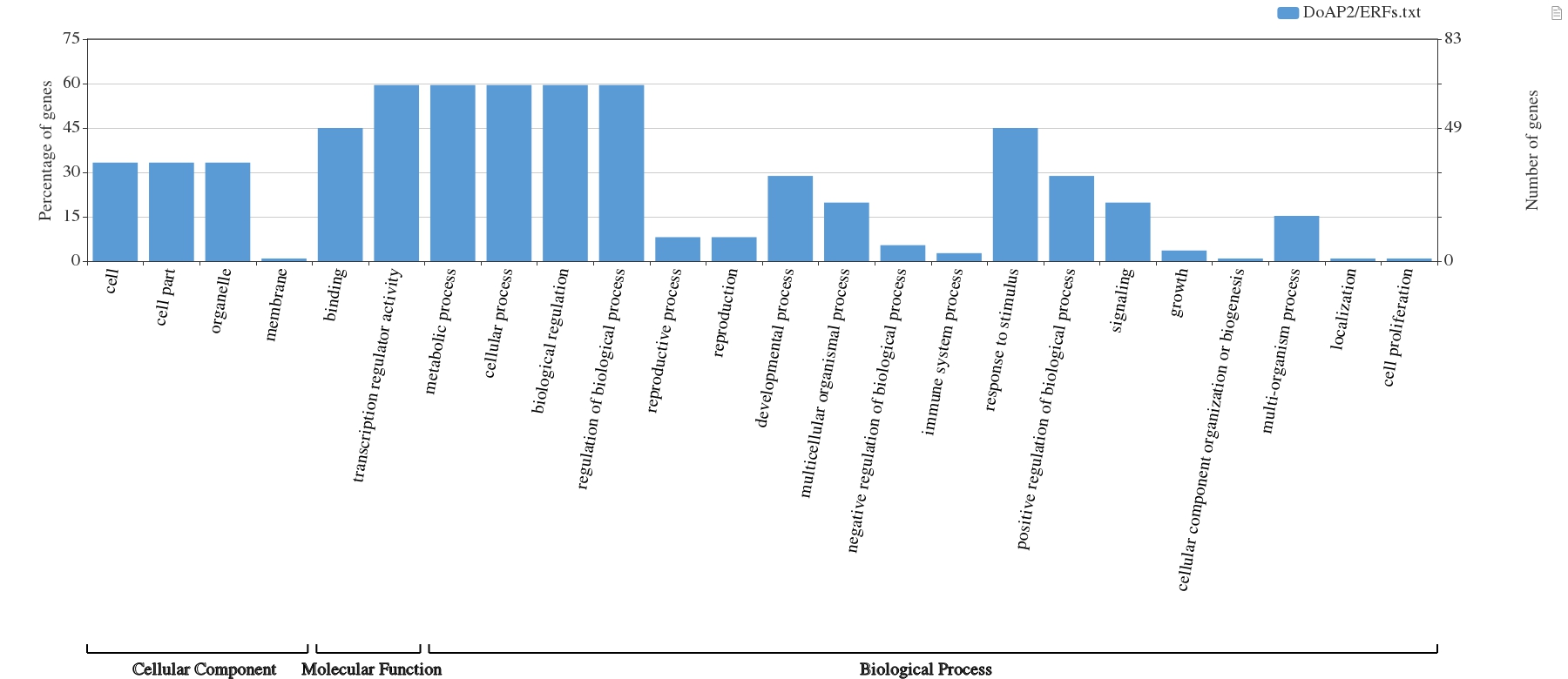

Supplement: Supplemental Information 4 — The 111 DoAP2/ERFs protein sequences are divided into three categories based on amino acid similarity, namely cellular components (CC), molecular function (MF), and biological processes (BP). [file peerj-12-16760-s004.jpeg]

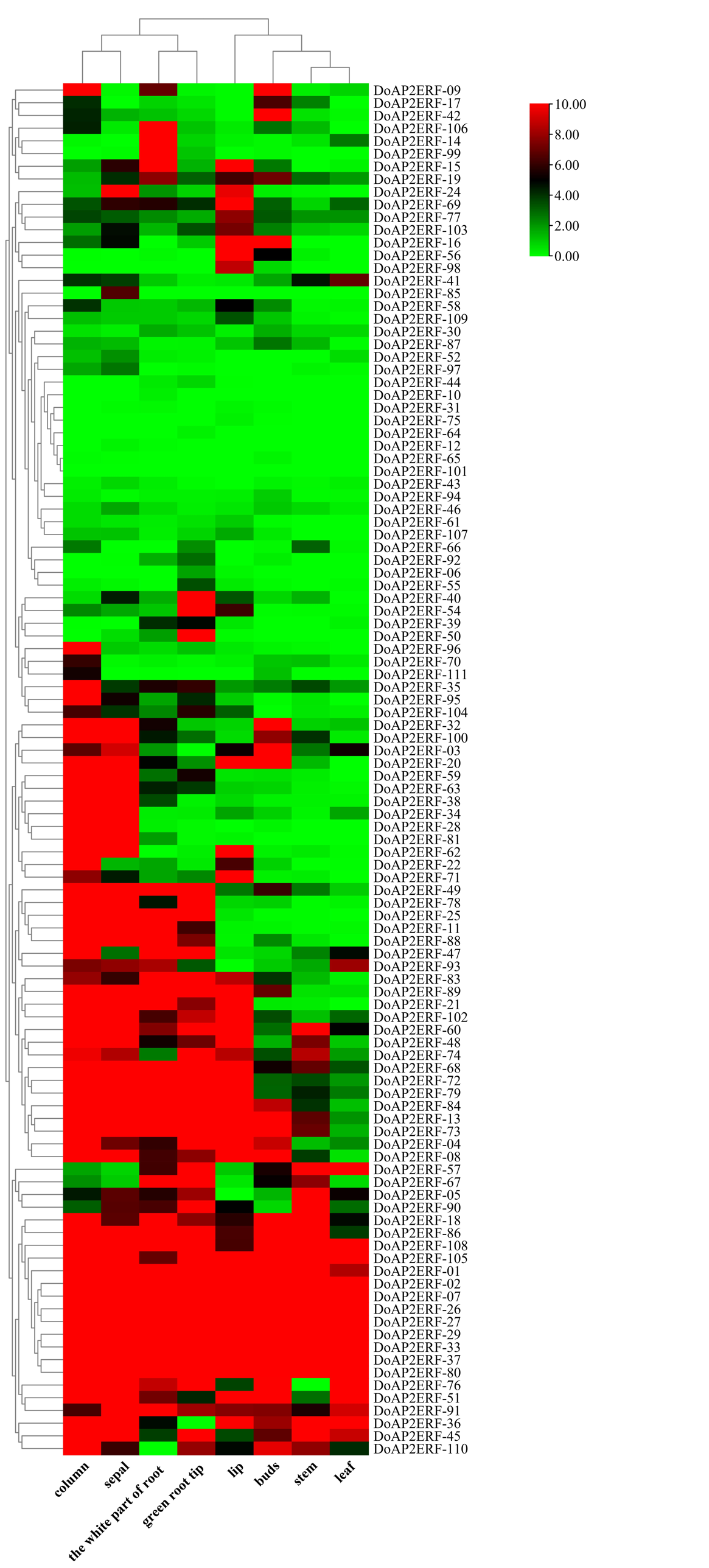

Supplement: Supplemental Information 5 — The heatmap was generated using TBtools and the FPKM values of D. officinale genes were evaluated and normalized based RNA-seq data from NCBI SRA database. Differential expression pattern of 111 annotated DoAP2/ERFs in various tissues, including roots, root tips, stems, leaf, lip, buds, and sepal. [file peerj-12-16760-s005.jpg]
